# Giving Broiler Feathers a Good Fate. Optimization of Chemical Hydrolysis for the Production of a Hydrolysate Rich in Free Amino Acids

**DOI:** 10.1021/acsomega.5c01262

**Published:** 2025-06-03

**Authors:** Victor E. Broering, Patrícia M. Stuelp Campelo, Pedro V. Michelotto, Luiz F. Bianchini, Edvaldo A. R. Rosa

**Affiliations:** † Chemical Engineering Faculty, UniFacvest University Centre, Av. Mal. Floriano, 947-Centro, CEP 88503-190 Lages, Brazil; ‡ Graduate Program in Animal Science, 28100 Pontificia Universidade Catolica do Parana, Xenobiotics Research Unit, 1155 Imaculada Conceicao St, 80215-900 Curitiba, Parana, Brazil; § Pontificia Universidade Catolica do Parana, Xenobiotics Research Unit, 1155 Imaculada Conceicao St, 80215-900 Curitiba, Parana, Brazil

## Abstract

The poultry meat production chain generates considerable waste, especially feathers. Feathers, composed of low solubility, digestibility, and chemical stability keratins, pose a significant challenge for reuse. This study, however, has successfully optimized the alkaline hydrolysis process using sodium hydroxide, potassium hydroxide, and an equimolar combination under varying temperature and time conditions. The different hydrolysis conditions were statistically evaluated to identify critical optimization points. The errors between the predicted and experimentally acquired results were remarkably low, providing a high level of reliability for the process. They achieved higher protein hydrolysis rates (97.9% for NaOH, 96.7% for KOH, and 97.8% for the mixture). Analysis by ^13^C nuclear resonance (^13^C NMR) spectroscopy was conducted on both intact feather samples and obtained hydrolysates, demonstrating the efficiency of breaking down keratin into free amino acids and peptides. The merit of this study is not just in its successful demonstration of a high hydrolysis of the keratin in broiler feathers from industrial abattoirs using sodium hydroxide (a low-cost alkali) at a low concentration (2.6%) and a temperature of 75.6 °C, but also in its potential to provide a sustainable solution for the waste management and poultry meat production industries. The results show their possible applicability on an industrial scale, as the resulting optimal conditions are mild.

## Graphical abstract

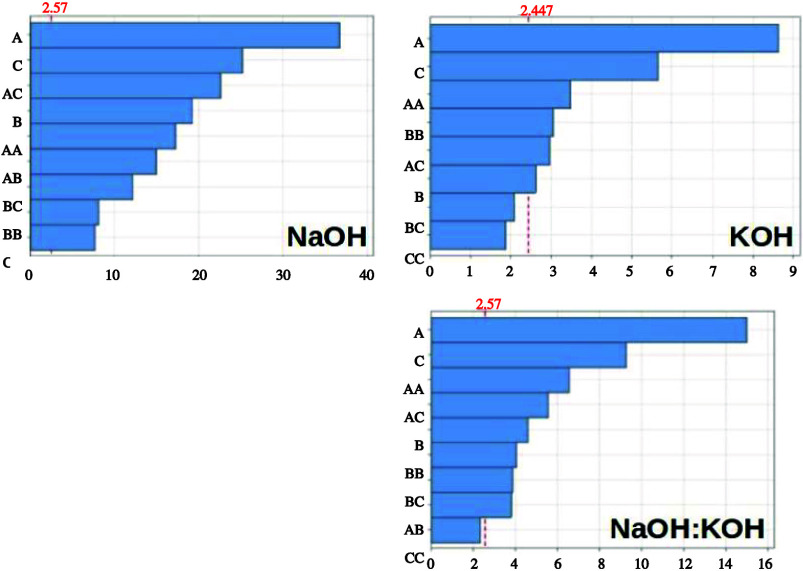

## Introduction

During broiler meat processing, much biogenic waste is generated, especially feathers. Feathers account for approximately 5 to 7% (mean = 6%) of a bird’s total mass,^1,2^ assuming that an average bird weighs 2.8 kg at 45 days old.^3^ Global broiler production in 2021 exceeded 33 billion birds;^4^ it can be inferred that over 5.544 million tons of broiler feathers are industrially generated annually.

Due to their low density (∼0.8 g cm^–3^)^5^ and structural architecture, the volume occupied by residual feathers in the processing becomes a logistical problem for slaughterhouses and poultry plants. Their disposal usually occurs through incineration or composting,^6^ which generates numerous environmental issues and results in the wastage of raw materials, even though feathers are considered end-of-life (EoL) products.^7^

The main component of feathers is keratin (>90%),^8^ arranged as β-pleated sheets and rich in hydrophobic amino acids such as alanine, valine, leucine, isoleucine, methionine, tryptophan, and phenylalanine. The sulfur-containing polar amino acid, cysteine, contributes to the high stability of the structure through the formation of covalent disulfide bonds.^2^

Due to its chemical and structural characteristics, keratin exhibits low solubility, high resistance to enzymatic action, and low digestibility in vertebrates, with only a few microorganisms capable of breaking down its structure and absorbing its amino acids.^9^

However, keratin hydrolysates are products of great interest in various industries. They are the main components of feather meals, with high nutritional potential for animal feed formulation.^10,11^ They are adhesive binders used in the furniture industry to produce particleboard panels.^12^ They exhibit considerable biosorption properties for heavy metals and can be used in bioremediation processes.^13^ Additionally, they function as moisturizing agents for skin and hair and can be incorporated into various cosmetic products.^14^ Furthermore, they can be converted into biomethane.^15^

The hydrolysis of feather keratin involves simultaneous steps of protein denaturation, breaking of disulfide bonds, and peptide bond cleavage, leading to the release of amino acids. This process can be achieved using hydrolytic enzymes under mild conditions or denaturing substances that significantly modify pH or redox potential (*E*_h_), using high temperatures for several hours.

Less costly processes involve using alkali metal hydroxides like sodium and potassium hydroxides or alkaline earth metal hydroxides like calcium hydroxide.^16–19^ Increasing the temperature promotes protein denaturation and weak interaction destabilization, such as hydrogen bonds. Disulfide bridges are broken during hydrolysis, followed by the breakdown of primary protein structures, releasing short peptides and amino acids. Chemical hydrolysis can also be facilitated using denaturing agents such as detergents (sodium dodecyl sulfate), reducing agents (sodium bisulfite, sodium sulfite, and sodium thioglycolate), solutes (urea, guanidine hydrochloride, hydrophobic ionic liquids), individually or in combination, but at a higher cost.^20–22^

Several groups have been striving to reduce the costs of obtaining feather keratin hydrolysates to make their production more viable, enhance their utilization, or lessen the environmental impact of production within a “Green Production” philosophy.^16,23–26^ However, the results vary significantly, and only a few variables that make up the hydrolysis cost are evaluated per article.

This study aimed to optimize the chemical hydrolysis process of feather keratin using NaOH, KOH, or an equimolar combination by varying their concentrations, process temperature, and hydrolysis time. The goal was to achieve the optimal procedure to run hydrolysis with the minimum energy input and chemical usage, thus making the process viable on an industrial scale.

## Materials and Methods

### Origin and Pretreatment of Feathers

Feathers from a commercial hybrid Cobb-Vantress broiler collected at an industrial slaughterhouse in Alto Vale do Itajaí, Brazil, were used. The feathers were washed thrice by immersion in water at 60 °C for 60 min to remove blood, feces, and skin residues. Subsequently, they were immersed in a 0.5% (v/v) sodium hypochlorite solution for 12 h for disinfection. Afterward, they were rinsed three times with running water, centrifuged, and dried in an oven at 60 ± 4 °C.^22^

The feathers were ground using a Wiley R-TE-650/1 knife mill (Tecnal Ltd., Piracicaba, Brazil). The ground material was classified to a size of ≤5 mm using an ABME-0800 sieve shaker (Bronzinox Ltd., São Paulo, Brazil). The fragmented feathers were vacuum-packed and kept at −18 °C.

The Kjeldahl method was used to determine the total nitrogen and calculate the protein content of the feather samples.^27^ The sample digestion was done using a sample digester block TE-005/50-GE (Tecnal Ltd., Piracicaba, Brazil) with concentrated sulfuric acid and a catalytic mixture at 100 °C for 30 min. After this period, the temperature increased in 50 °C intervals every 30 min until reaching 350 °C, then maintained at this temperature until a greenish-blue color was obtained (3–4 h).

The distillation was carried out with the digested sample, cooled to room temperature, using boric acid, 50% NaOH, and a nitrogen distillatory TE-0364 (Tecnal Ltd., Piracicaba, Brazil). The titration step was performed with previously standardized 0.1 M HCl using a digital buret Titratte (Brand GmbH + Co KG, Wertheim, Germany) until the solution’s color changed based on the indicator used, indicating the end point of the titration. The content of Total Nitrogen as a mass percentage (%NT) in the feather samples was calculated using eq 1. In the equation, *V* is the volume of HCl solution used in the titration (mL), *M* is the theoretical molarity of the HCl solution, *f* is the correction factor of the HCl solution, and *P* is the mass of the sample (g).

1 %NT=[(V×M×f×0.014)/P]×100

The protein content (%) was determined by multiplying the Total Nitrogen Content (%NT) by the value of the Protein Conversion Factor (6.25) described in the literature (eq 2). This conversion factor is based on the premise that, on average, nitrogen corresponds to 16% of the total protein weight in foods and food samples of animal origin, including animal feeds.^27^ The protein content was 93.61 ± 0.03.

2 %protein=%NT×proteinconversionfactor(6.25)

In this work, feather protein was determined to calculate the yield of the chemical hydrolysis process, providing data for estimating the ability to transform crude protein into free amino acids in the hydrolysate.

All determinations were performed in triplicate, and values were presented as mean value ± standard deviation.

### Experimental Design and Statistical Analysis

A Box–Behnken experimental design (BBD) was employed to optimize the hydrolysis conditions with three factors and three experimental levels each. The factors were temperature, hydrolysis time, and hydroxide concentration. These factors were coded at three levels: minimum (−1), central point (0), and maximum (+1), with median distributions between each level.^28,29^ Table 1 presents the design of the factors and levels established in the BBD.

**Table 1:** Factors and Experimental Levels Established for the Box–Behnken Experimental Design

	levels		
factors	minimum value (−)	central point (0)	maximum value (+)
temperature (*T*; °C)	50	70	90
time (*t*; min)	60	180	300
concentration (%)	1	2	3

The total number of experiments was calculated using eq 3. In the equation, *N* is the total number of experiments, *k* is the number of variables, and *C*_0_ is the number of repetitions of the central point (CP). This resulted in 15 independent experiments, with the central point in triplicate,^22,30^ as shown in Table 2.

3 $$N=2k\left(k-1\right)+C_{0}$$

**Table 2:** Box–Behnken Experimental Design Matrix^a^

	factors		
experiment	*T* (°C)	*t* (min)	concentration (%)
1	50 (−)	60 (−)	2 (0)
2	90 (+)	60 (−)	2 (0)
3	50 (−)	300 (+)	2 (0)
4	90 (+)	300 (+)	2 (0)
5	50 (−)	180 (0)	1 (−)
6	90 (+)	180 (0)	1 (−)
7	50 (−)	180 (0)	3 (+)
8	90 (+)	180 (0)	3 (+)
9	70 (0)	60 (−)	1 (−)
10	70 (0)	300 (+)	1 (−)
11	70 (0)	60 (−)	3 (+)
12	70 (0)	300 (+)	3 (+)
13 (CP)	70 (0)	180 (0)	2 (0)
14 (CP)	70 (0)	180 (0)	2 (0)
15 (CP)	70 (0)	180 (0)	2 (0)

a CP= central point.

The evaluated alkalis were sodium hydroxide (NaOH), potassium hydroxide (KOH), and a NaOH/KOH mixture in a 1:1 w/w ratio. Each alkali was used in a separate optimization process, generating three independent response surfaces.

Analysis of variance (ANOVA) was used to determine the variables with statistically significant effects on the evaluated response. The best extraction conditions (optimal point) were determined using the response surface methodology (RSM),^31,32^ utilizing R software version 4.2.1 (R Core Team, Vienna, Austria).

### Hydrolysis

The experiments used 15 g of ground feathers mixed with 150 mL of hydrolysis solutions (distilled water and hydroxides) in 500 mL Erlenmeyer flasks with ground glass necks. Liebig condensers of 300 mm were attached to prevent concentration by evaporation during hydrolysis. The setups were placed in a temperature-controlled water bath. The timer was started once the designated temperature for a specific treatment was reached. After the specified experiment time, the setups were removed from the water baths and allowed to cool while keeping the condensers attached and circulating water.

Once the postreaction products (hydrolysates) reached room temperature, the contents were transferred to 50 mL polypropylene tubes and centrifuged at 3860*g* for 10 min using a K14–5000 M centrifuge (Kasvi Ltd., São Jose dos Pinhais, Brazil). The supernatants were then transferred to clean tubes.

### Determination of Total Nitrogen

The Kjeldahl method^27^ was used to determine the hydrolysates’ Total Nitrogen (%NT).

### Determination of Amino Acids by Nuclear Magnetic Resonance (^13^C NMR)

The protein hydrolysates and ground feathers were subjected to the qualitative determination of the amino acids serine, cysteine, leucine, valine, and proline by carbon-13 nuclear magnetic resonance (^13^C NMR). The samples were dried under Argon and then dissolved in deuterated water (D_2_O) to a concentration/dispersion of 21 mg mL^–1^. The solutions/dispersions were filtered and analyzed using a Bruker Avance 500 MHz ^13^C nuclear magnetic resonance spectrometer (Bruker Co, Karlsruhe, Germany). The obtained spectra were quantified using Mnova software version 14.3.2 (Mestrelab Research, Santiago de Compostela, Spain).

## Results and Discussion

The Pareto diagrams below (Figure 1) show the absolute and standardized effects’ values and interactions in descending order, with the vertical line indicating which effects are significant. The reference line (vertical) depends on the significance level (α = 0.05). It is noticeable that in NaOH, all parameters and their interactions are significant, with temperature and alkali concentration showing a greater influence. On the other hand, in KOH, the interaction of time with the alkali and the quadratic alkali variable was not significant. Once again, temperature and alkali significantly influenced the response variable (%NT). The same behavior is observed in the mixture of temperature and alkali, with the quadratic alkali being nonsignificant. As the values on the diagram are absolute, we can determine the magnitude of the effects of each studied variable and interaction. Still, we cannot determine whether they cause an increase or decrease in the response variable. For this, the coefficients of the equations obtained in the study should be observed.

**Figure 1:**
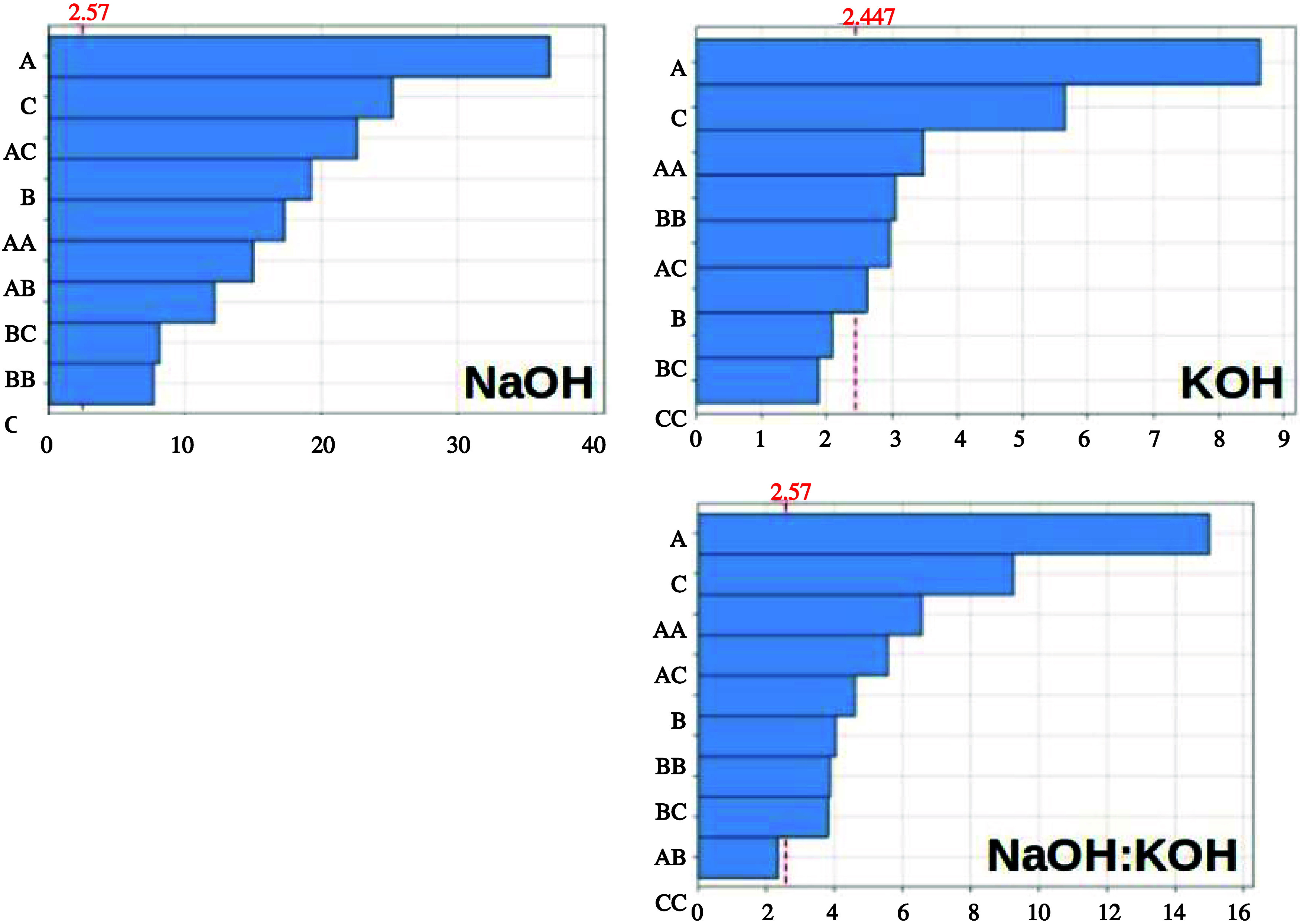
Pareto diagrams of the effects of the factors evaluated in the factorial design for the three hydrolyses. α = 0.05. In the *y*-axes: A = temperature; B = time; C = concentration.

The analysis of the effects of the factors involved in the design, with 95% confidence (α = 0.05), showed that factors A (temperature), B (time), and C (concentration) were significant in all evaluated models, with temperature and alkali concentration showing a more substantial influence on the response (%NT). Denaturing agents such as temperature and alkalis promote the breakdown of the protein structures of keratin, contributing to its destabilization. Thus, using these factors as control and their strong influence on the model are justified in feather hydrolysis.^18,19,22^

Table 3 presents the values obtained from analyzing quadratic models of the hydrolysis processes. The coefficient of determination (*R* ^2^) indicates the proportion of total variation explained by the model, and the results obtained can be considered adequate, with values ranging from 0.9613 to 0.9986. Values close to 1 indicate a good fit of the model to the data.

**Table 3:** Parameters Obtained for the Quadratic Models of the Three Hydrolyses

	NaOH	KOH	NaOH/KOH
mean	0.9880	0.9047	0.9260
mean deviation	0.0236	0.1362	0.0749
*R* ^2^	0.9986	0.9613	0.9889
*R*^2^ predicted	0.9812	0.6084	0.8342
*R*^2^ adjusted	0.9962	0.9096	0.9690

Adjusted *R* ^2^ values indicate model adequacy more precisely as they penalize nonsignificant parameters and terms added to the model (except in hierarchical cases). Unnecessary parameters can lead to an increase in *R* ^2^ without necessarily improving the model. Adjusted *R* ^2^ is more reliable for comparing models with different numbers of parameters. In this case, adjusted *R* ^2^ values ranged from 0.9096 to 0.9962. Therefore, the model obtained by RSM for KOH explains 90.96% of the variability in the response, in this case, the percentage of acquired nitrogen. For NaOH, it explains 99.62%, and for the mixture, it explains 96.90%. These values alone do not guarantee a flawless model, but when combined with other analyses, they represent a good value for the adjusted coefficient of determination. Considering only the adjusted *R* ^2^, the most appropriate model would be the one using NaOH.

The model evaluation was performed using analysis of variance (ANOVA) for each response, and the effects were verified through p-values with a confidence level of 95%. Additionally, lack of fit was evaluated because an appropriate model should have a significant regression and a nonsignificant lack of fit (preferably with p-values greater than 0.1).

The Response Surface Methodology (RSM) was used to obtain the quadratic models of the experiments and their respective optimum points (Figure 2). The equations obtained are presented in Table 4. The equations are coded (standardized) with the variables, allowing for the examination of the influence of the independent variables, their quadratic forms, and their respective interactions. In the study with KOH, the interaction between temperature and time was insignificant and, therefore, removed from the equation.

**Figure 2:**
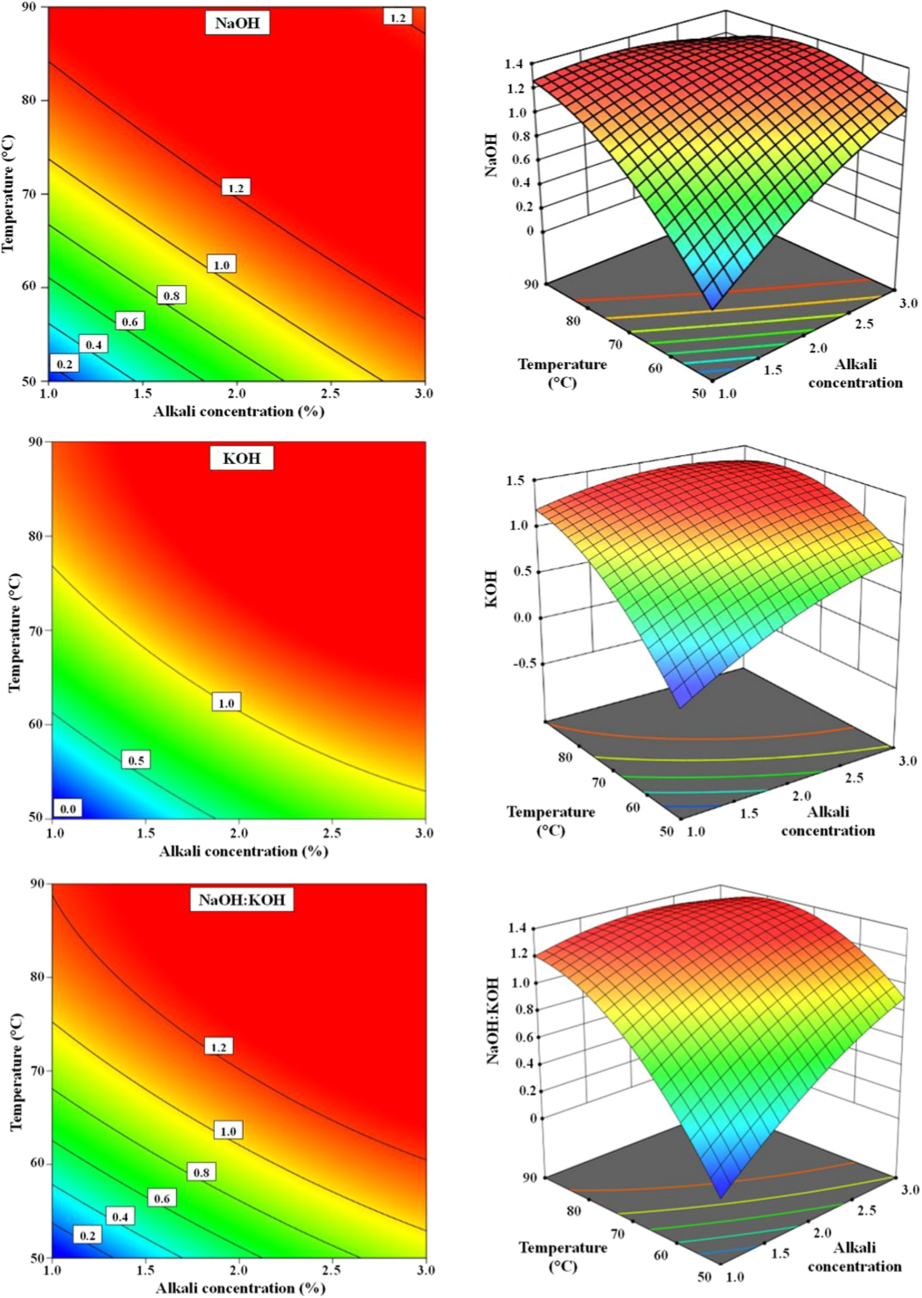
Response surfaces and contour plots showing the effects of temperature and alkali variables for the hydrolysis performed.

**Table 4:** Equations Obtained after the Analysis of Experiments with RSM^a^

treatment	equation
NaOH	$R=1.21+0.3075\times A+0.1612\times B+0.2113\times C-0.1775\times \mathrm{AB}-0.2675\times \mathrm{AC}-0.1450\times \mathrm{BC}-0.2133\times A^{2}-0.1008B^{2}-0.0958\times C^{2}$
KOH	$R=1.22+0.4162\times A+0.1262\times B+0.2725\times C-0.2025\times \mathrm{AC}-0.1425\times \mathrm{BC}-0.2467\times A^{2}-0.2167\times B^{2}-0.1342\times C^{2}$
NaOH/KOH	$R=1.20+0.395\times A+0.1212\times B+0.2438\times C-0.1425\times \mathrm{AB}-0.2075\times \mathrm{AC}-0.1450\times \mathrm{BC}-0.2558\times A^{2}-0.1583\times B^{2}-0.0933\times C^{2}$

a A = temperature; B = time; C = alkali.

The response surfaces generated were used to indicate the optimum conditions for the chemical hydrolysis of feathers using the experimental matrix described in Table 2. The optimum point (critical point) is obtained using the equations obtained from the surfaces. As the goal is to optimize the alkaline hydrolysis process to obtain an amino acid-rich solution, the point, in this case, represents a maximum value. Thus, the end of interest is the one that zeros the partial derivatives of each of the involved variables, and the guarantee that the obtained critical points are maximum comes from a canonical analysis where all eigenvalues are negative. This analysis extracted the temperature, time, and alkali percentage (Table 5), resulting in the maximum value (%NT optimization). These values indicate the ideal conditions for nitrogen extraction, avoiding unnecessary or insufficient use of temperature, time, or alkali. They present the perfect relationship between them.

**Table 5:** Critical Point Values with the Expected and Obtained %NT (Total Nitrogen) Values and Their Respective Errors

treatment	temperature (°C)	time (min)	alkali (%)	%NT expected	%NT obtained	error (%)	protein obtained (g)	yield (%)
NaOH	75.6	191	2.6	1.330	1.302	2.1	13.74	97.9
KOH	83.3	197.8	2.4	1.338	1.294	3.3	13.57	96.7
NaOH/KOH	78.4	144.6	3	1.328	1.298	2.2	13.72	97.8

Since these models are purely statistical studies, they do not have a limitation concerning stoichiometry, i.e., the amount of matter in the specific experiment. Therefore, to adjust the analysis to the presented reality, a limitation was added to the optimization: the composition of the feather itself. In the physicochemical characterization of the feathers, 93.61 ± 0.03% of protein was obtained, representing 14.04 g of protein per 15 g of feathers used. Using these values, the maximum value of nitrogen obtained for each alkali used is determined, representing the expected value in the hydrolysis process. Afterward, these points underwent experimental tests, calculating the errors between the expected and obtained %NT (Table 5).

The combination of temperature, concentration, and alkali at the studied values showed that it is possible to hydrolyze the feather mass almost entirely with high yield values (Table 5). This can be observed through the hydrolysis yield formula (eq 4).^25^ Using these parameters ensures an energy-saving advantage compared to previously recommended protocols, which require prolonged digestion times, higher alkali concentrations, or even using other reagents.

4 y[%]=(total protein obtained hydrolysis(g)/total protein(g))×100

Using alkali to hydrolyze feathers’ keratin is not necessarily a new approach; however, few groups have prospected industrial feasible protocols. We aimed to improve the process to allow for less alkali (and further acid to neutralize it), less energy input, and less time. The results revealed that using NaOH (an inexpensive alkali) at a low concentration (2.6%) and a temperature of 75.6 °C in three hours, it can obtain 97.9% hydrolysis efficiency. Similar published studies that evaluated fewer parameters have obtained inferior yields.^25,33,34^ Other proposed technologies increase the costs because they use expensive enzymes,^35,36^ autoclaving/microwaving steps,^37,38^ or steam-flash explosion treatment,^39,40^ and some result in yields inferior to those obtained here.

The ^13^C nuclear magnetic resonance (NMR) spectroscopy analysis was conducted with treated and ground feather samples. The hydrolysates were obtained after hydrolysis with the three alkalis at their optimum points.

This analytical tool demonstrates the presence of free amino acids in the protein hydrolysates, confirming the breakdown of the keratin structure.^41–43^

^13^C NMR spectroscopy uses theoretical chemical shifts (in δ [ppm]) to identify carboxyl, amino, radical, and hydrogen functional groups linked to the asymmetric and chiral α-carbon (α-C) of the amino acids (Table 6). The indicator amino acids for confirming hydrolysis were cysteine, leucine, proline, serine, and valine, common in keratins.

**Table 6:**
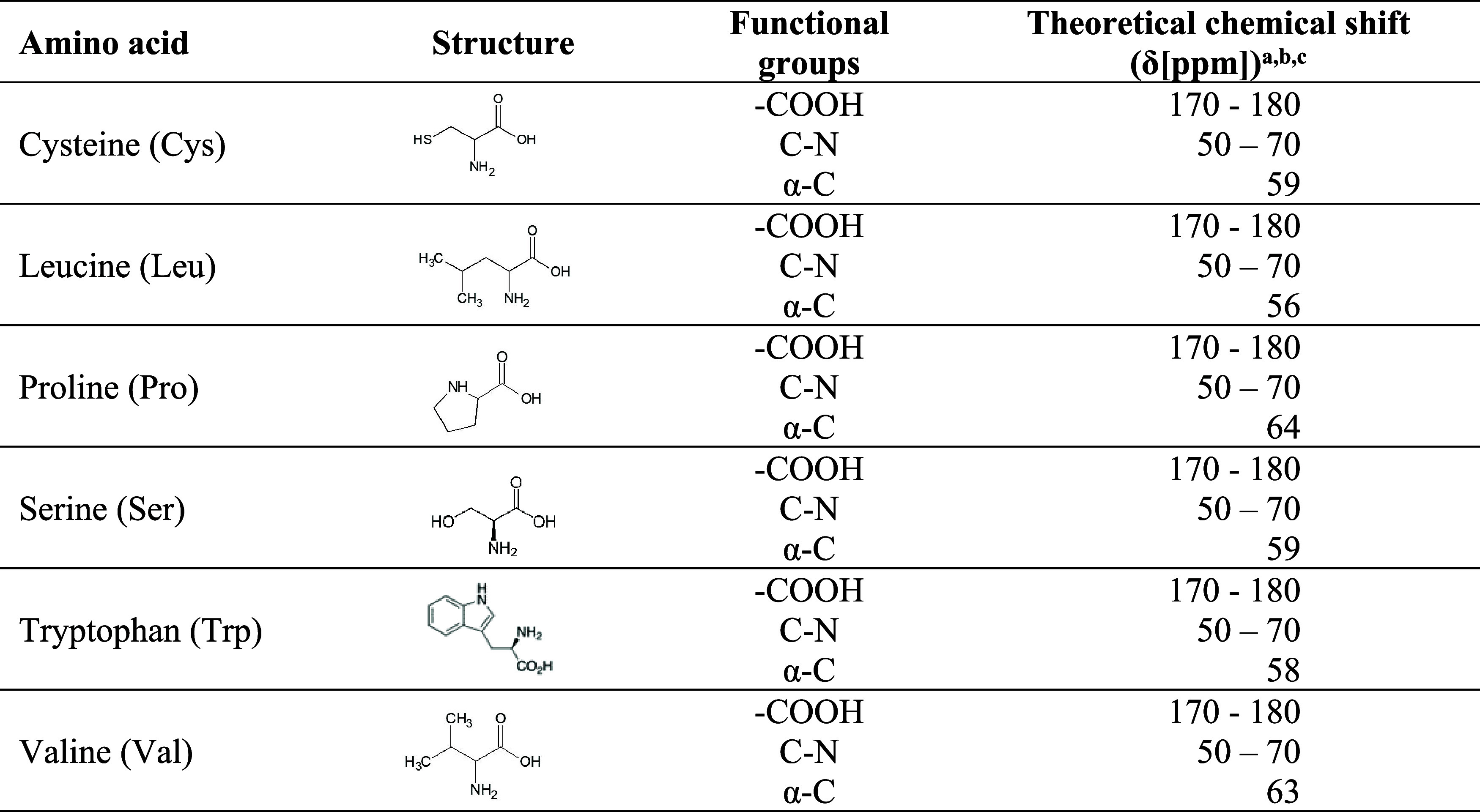
Structure of Monitored Amino Acids by ^13^C NMR in the Samples, with Functional Groups and Respective Chemical Shifts^a^

a a = CHAPMAN et al., 1994; b = SILVERSTEIN e WEBSTER, 2000; c = KENNEPOHL, 2023.

Figure 3 shows that the spectra obtained for untreated and ground feathers did not exhibit characteristic shifts of the evaluated amino acids. This observation confirms that there are no free amino acids in this form of presentation, indicating the maintenance of keratin’s structural integrity.

**Figure 3:**
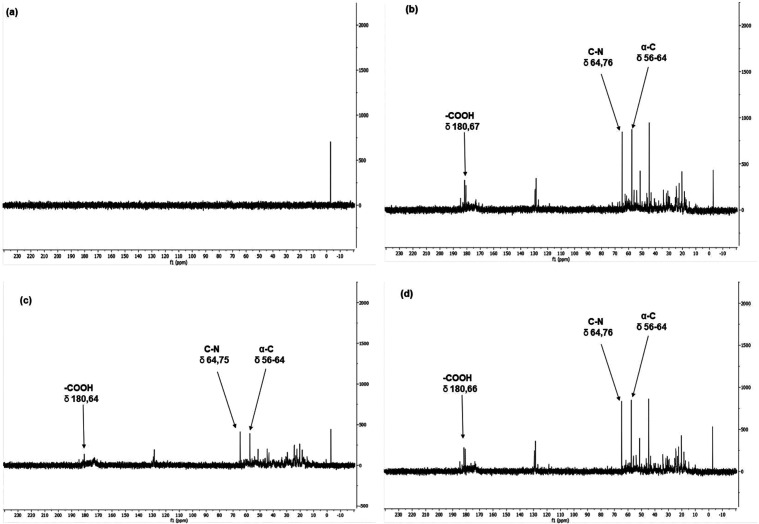
^13^C NMR spectra of crude feather sample (a) and protein hydrolysates (b = NaOH, c = KOH and d = NaOH/KOH).

On the other hand, the spectra of the hydrolysates revealed indicative peaks in the monitored chemical shift ranges, indicating the presence of carboxyl functional groups, carbon atoms linked to the nitrogen of amino groups (C–N), and typical α-C shifts of the monitored amino acids (Table 6).^44^

Alkaline hydrolysis can be slower and somehow incomplete compared to acid hydrolysis. However, unlike the second, alkaline hydrolysis has a lower loss of amino acids, such as tryptophan.^15^ This amino acid is necessary for maintaining muscles and producing various bioactive compounds such as serotonin, melatonin, nicotinamide, etc. Tryptophan undergoes degradation under acidic conditions.^45^ The ^13^C NMR spectrum shows strong evidence of the presence of tryptophan as a product of the hydrolysis performed in this study. However, other analysis methods are needed to corroborate the evidence. In the case of proof, the result of hydrolysis becomes even more critical due to the high added value of this amino acid.

It is important to emphasize that, still in the initial phase of this study, the possibility of using Ca­(OH)_2_ in hydrolysis was considered, as suggested by some authors.^17,18^ However, even using such alkali at 3%, at 90 °C, and for 300 min, hydrolysis resulted in merely 37.13% of feather mass digested. This result contrasted with those obtained with the other treatments, letting us discard such a possibility before proceeding with the optimization experiments. It was considered that to get satisfactory results, Ca­(OH)_2_ hydrolysis would demand harsher conditions, incurring increments of alkali amounts, time and energy input. As the objective of this study was to establish cleaner and more economical options for an industrial scale-up, it was decided not to include Ca­(OH)_2_ in subsequent steps.

In addition to the amino acids obtained, alkaline hydrolysis results in nonreactant NaOH, which must be neutralized. This can be done with acetic acid if the final destination is to use feather-derived amino acids for biomethanization^15^ or orthophosphoric acid if the intention is to use them as plant fertilizer/biostimulant.^26^ Such nonreactant NaOH can hydrolyze more feather mass if the solid-to-liquid ratio increases.

## Final Considerations

This study demonstrated that it is possible to conduct high hydrolysis of the keratin in broiler feathers from industrial abattoirs using NaOH (a low-cost alkali) at a low concentration (2.6%) and a mild temperature of 75.6 °C. Other studies have proposed using NaOH for feather hydrolysis. However, they involved higher alkaline concentrations and temperature inputs or demanded more time to furnish amino acids, peptides, or protein fragments. Here, we parametrized all the variables using robust experiment design tools, and the proposed protocol allowed the maximum free hydrolysis products to be obtained with minimum energy input and time. Allied with the achieved higher protein hydrolysis (97.9%), this combination of factors makes our protocol of alkaline hydrolysis attractive from an industrial perspective, and it may favor its adoption, reducing the environmental impact of feather disposal/incineration and creating added value to this underestimated byproduct.
